# Health insurance status, lifestyle choices and the presence of non-communicable diseases: a systematic review

**DOI:** 10.1093/pubmed/fdad247

**Published:** 2023-12-11

**Authors:** Adeola Folayan, Mark Wing Loong Cheong, Quek Kia Fatt, Tin Tin Su

**Affiliations:** South East Asia Community Observatory (SEACO), Jeffrey Cheah School of Medicine and Health Sciences, Monash University Malaysia, 47500 Bandar Sunway, Malaysia; School of Pharmacy, Monash University Malaysia, 47500 Bandar Sunway, Malaysia; Global Public Health, Jeffrey Cheah School of Medicine and Health Sciences, Monash University Malaysia, 47500 Bandar Sunway, Malaysia; South East Asia Community Observatory (SEACO), Jeffrey Cheah School of Medicine and Health Sciences, Monash University Malaysia, 47500 Bandar Sunway, Malaysia

**Keywords:** systematic review, health insurance status, lifestyle choices, non-communicable diseases

## Abstract

**Background:**

Although health insurance (HI) has effectively mitigated healthcare financial burdens, its contribution to healthy lifestyle choices and the presence of non-communicable diseases (NCDs) is not well established. We aimed to systematically review the existing evidence on the effect of HI on healthy lifestyle choices and NCDs.

**Methods:**

A systematic review was conducted across PubMed, Medline, Embase, Cochrane Library and CINAHLComplet@EBSCOhost from inception until 30 September 2022, capturing studies that reported the effect of HI on healthy lifestyle and NCDs. A narrative synthesis of the studies was done. The review concluded both longitudinal and cross-sectional studies. A critical appraisal checklist for survey-based studies and the National Institutes of Health Quality Assessment Tool for Observational Cohort and Cross-Sectional Studies were used for the quality assessment.

**Result:**

Twenty-four studies met the inclusion criteria. HI was associated with the propensity to engage in physical activities (6/11 studies), consume healthy diets (4/7 studies), not to smoke (5/11 studies) or take alcohol (5/10 studies). Six (of nine) studies showed that HI coverage was associated with a lowered prevalence of NCDs.

**Conclusion:**

This evidence suggests that HI is beneficial. More reports showed that it propitiated a healthy lifestyle and was associated with a reduced prevalence of NCDs.

## Introduction

Non-communicable diseases (NCDs) include four disease clusters: cardiovascular diseases, diabetes, cancer and chronic obstructive pulmonary diseases.^[^^1–4^^]^ These clusters contribute to the highest morbidity burden globally^4^ and are the leading causes of premature mortality worldwide.^4–6^ They account for 73% of mortality deaths globally,^7^ of which most reported mortality could be prevented.^3^^,^^7^ NCDs are linked to four lifestyle risk factors: tobacco smoking, poor diet, physical inactivity and alcohol consumption.^4^^,^^8^^,^^9^ A healthy lifestyle has been known to reduce the risks of developing NCDs.^10–13^ A cohort study by Lv *et al.*^12^ among Chinese adults has shown that about three out of four incidences of Type 2 diabetes could be avoided in <10 years with adherence to a healthy lifestyle. In a similar study in China, non-smoking, moderate alcohol intake, physical activity and a healthy diet were associated with lower risks of major coronary events and ischemic stroke.^11^

Health insurance (HI) is a means to finance health care expenses by risk and resource pooling,^14^^,^^15^ which is more likely to be efficient and equitable than the ‘out-of-pocket’ health payment method.^14^ The effect of HI on healthy lifestyle choices and health outcomes is not well established to date. The available evidences are inconclusive. Some studies claim HI increases the propensity to engage in a healthy lifestyle,^16–18^ while others claim HI decreases the propensity to engage in a healthy lifestyle.^19^^,^^20^ In contrast, some studies found that HI does not affect healthy lifestyle choices and health outcomes.^21^^,^^22^

However, there is a risk that HI may induce Ex-ante moral hazard. Ex-ante moral hazard occurs when HI reduces an individual interest in protective behaviours. Ex-ante moral hazard is not an intended outcome of HI^23^ but a possible outcome of HI.^13^^,^^24^ On the other hand, there could be a case of propitious selection where an individual purchases HI and still engages in a healthy lifestyle.^1^ This is a likely situation for people with high risk-averse who would like to avoid risk as much as possible. Ex-ante moral hazard could induce unhealthy lifestyles, precipitate NCDs related risk factors and subsequently lead to the incidence of NCDs. Considering the link between a healthy lifestyle and the incidence of NCDs, it is important to understand the effect of HI on lifestyle.

The primary aim of this systematic review is to review and summarize research that examined the effect of HI on healthy lifestyle choices and the presence of NCDs. This is to establish if HI is beneficial for healthy lifestyle choices and outcomes related to NCDs. This will provide information that can be used to develop effective policy reforms that can optimize HI plans.

## Methods

The Preferred Reporting Items for Systematic Reviews and Meta-Analyses guidelines were followed for the systematic review. The protocol (Identification Number: CRD42021236377) was registered with the International Prospective Register of Systematic Reviews. Relevant studies were collected through electronic databases using predefined search terms and synonyms.

The search strategy is detailed in Appendix A. The following databases were searched: PubMed, Medline, Embase, Cochrane Library and CINAHLComplet@EBSCOhost. Alerts were created on the databases to notify new articles related to the search terms before the final analysis. Reference lists of eligible articles were searched for relevant literature.

### Eligibility criteria

The established inclusion and exclusion criteria were used to screen identified articles for the review. The following study parameters were used to decide which studies to include: (i) population: studies that examined the adult population (from 18 years of age), (ii) intervention: studies that examine the effect of any HI scheme on at least one of the below outcomes, (iii) comparison: studies that compared HI with no insurance or other forms of insurance for studies with control conditions and (iv) outcome variables: the four major NCDs (cardiovascular diseases, cancer, chronic respiratory diseases and diabetes) and four major lifestyle-related risk factors (tobacco smoking, alcohol consumption, poor diet and physical inactivity identified by the World Health Organization^25^).

Eligible studies included case–control, cohort studies, cross-sectional surveys and studies that reported quantitative data on the effect of HI schemes on at least one of the aforementioned outcome variables. The review included all studies from the inception of the selected databases until 30 September 2022. Only English articles were included. Abstracts, presentations, conference proceedings, unpublished data, letters to the editor, editorials and comments were excluded to avoid duplicate data and data that had not undergone peer review.

### Data extraction

All searches were done by two reviewers (AF and MC). A third reviewer (TTS) was consulted on any disagreement. The selection of articles was conducted in three steps. In the first phase, articles were extracted from the selected database. Secondly, articles with titles not relevant to the study were excluded after screening for duplicates. In the third phase, articles were assessed for eligibility.

Endnote X6 was used to save and organize details of studies and screened for duplicates. The collected data were summarized into an extraction table. Whenever necessary, the authors of the included articles were contacted for more information. Five authors were contacted and three authors responded.

### Quality assessment

A critical appraisal checklist for survey-based studies was used to assess the quality of the cross-sectional studies.^26^ The National Institutes of Health Quality Assessment Tool for Observational Cohort and Cross-Sectional Studies was used to assess the longitudinal studies.^27^ Only good and fair (medium) quality studies were included in the study. The two assessment tools are attached in Appendix B. The quality assessment procedure is in Appendix C. The quality assessment grade is shown in Table 1.

**Table 1:** Summary characteristics of included articles

Author and Publication date	Country	Objective	Study type	Control condition	Features of health insurance provided	Study period	Number of participant	Age of participant (Years)	Gender (female %)	Quality
**Aqtash and Servellen** ^17^	United States	To explore health-promoting lifestyle behaviours among Arab immigrants to the United States from the Middle Eastern region of the Levant	Cross-sectional, descriptive, non- experimental design				205	19–77	41.5	Good
**Axelrod** ***et al.***^35^	United States	To evaluate cardiac-metabolic burden by insurance status for Hispanic/Latino adults in Santa Barbara.	Cross-sectional survey	Insured versus uninsured	Any health insurance	July 2015–November 2017	593	18–85	73.2	Good
**Bittoni,** ***et al.***^39^	United States	To assess the effects of health insurance on cancer and chronic disease mortality, as well as the inter-relationships with diet, obesity, smoking and inflammatory biomarkers.	Prospective study	Private Insurance versus Public/no insurance		1988–1994	8950	>40	4645	Medium
**Castillo-** **Laborde** ***et al.***^40^	Chile	To assess Chilean public and private health insurance schemes’ performance and their effects on health inequalities.	Analytical observational study	National Health Fund versus private health insurance	Public/Private health insurance sub-system (FONASA/ISAPRE)	2013	16 657 500			Good
**Dave and kaestner** ^20^	United States	To explore the plausibly exogenous variation in health insurance as a result of obtaining Medicare coverage at age 65	Longitudinal study	Insured versus uninsured	Medicare	1992–2006	3396	63–64		Medium
**Decker,** ***et al.***^32^	United States	To document the health care needs and health risks of uninsured adults eligible for Medicaid	Cross-sectional survey	Medicaid enrolled versus uninsured	Medicaid	2007–2010	1513	19–64	55.1	Good
**Dong** ***et al.***^50^	China	To explore the impact of the Urban Resident Basic Health Insurance (URBMI) healthy behaviours and preventive health care services utilisation.	Longitudinal study	Publicly insured versus another publicly insured group	Urban Resident Basic Health Insurance versus Urban Employee Basic Medical Insurance	2006–2011	603	≥18	49.21	Medium
**Gotanda** ***et al.***^37^	United States	To examine the association bet- ween ACA-Medicaid expansion and changes in cardiovascular risk factors among the low income class.	Longitudinal study		Medicaid	2005– 2016	9711	19 to 64	54.4	Medium
**Hughes** ***et al.***^31^	United States	To examine the prevalence of health behaviours, clinical preventive services, and lifestyle risk behaviour among insured workers and examine health behaviour disparities based on demographic factors that exist among this group.	Cross- sectional analysis		Health insurance, prepaid plans (health maintenance organizations (HMOs) and Medicare).	2004– 2005	139 738 in 2004 and 159 755 in 2005	18 to 64	57.1 in 2005	Good
**Jerant** ***et al.***^22^	United States	To examine the association of health insurance change (gain or loss of coverage) with changes in preventive care and health behaviours	Longitudinal /Observa-tional study	Uninsured versus publicly insured versus privately insured	Publicly insured (Medicaid, and Medicare)-	2000– 2009	76 518	≥18	51.9	Medium
**Kim** ***et al.*** ^51^	South Korea	The study investigated the association between healthcare systems and arterial stiffness.	Cross- sectional study	Medical-aid versus National Health Insurance	National Health Insurance	January 2010- December 2016	8929	60	45.1	Good
**Mahoney** ***et al.***^52^	United States	To determine the associations between health insurance and undiagnosed diabetes and diabetes in a national sample of American adults.	Cross- sectional study	Insured versus uninsured	Medicaid, Private and Others	2005– 2016	13 029	18–64	49.6	Good
**Malta** ***et al.*** ^34^	Brazil	To compare the trend of risk and protective factors for NCDs in populations with and without health insurance	Popula- tion based cross- sectional study	Insured versus uninsured	Private health insurance.	2008– 2013	NA	≥ 18		Good
**Malta** ***et al.***^29^	Brazil	To analyse trends in risk and protective factors for non-communicable diseases (NCD) and access to preventive tests in the population with health insurance in Brazilian state capitals between 2008 and 2015.	Cross- sectional study			2008– 2015		≥ 18		Good
**Malta** ***et al.*** ^16^	Brazil	To describe health insurance coverage and compare the occurrence of risk factors and protective factors of non-communicable diseases in the population with and without health insurance in Brazilian state capitals.	Cross- sectional study	Insured versus uninsured		2015		≥ 18		Good
**Malta and Berna** ^28^	Brazil	To compares the risk and protective factors for Non-communicable Diseases (NCD), referred morbidity and access to preventive examinations in the population with and without health insurance	Cross- sectional popula-tion study	Insured versus uninsured		2011	54 099			Good
**Miraldo** ***et al*.** ^21^	United States	To determine the impact of expanding publicly subsidized health insurance through the Massachusetts reform on access to primary care, disease management and behavioural risk factors.	Cross-sectional survey		publicly subsidized health insurance through the Massachusetts reform	2001–2010	131 002	18–64	53.1	Medium
**Mpofu** ***et al.*** ^18^	Brazil	To estimate the prevalence of NCD risk factors and their associations with race, education, and health insurance status among non-pregnant women of reproductive age in Brazil.	Cross- sectional study	Insured ((≥ One private (prepaid group practice, medical cooperatives, and company health plans)) versus uninsured		2011	13 745	18–44	100	Good
**Qin and Lu** ^30^	China	To examine whether participating in the New Rural Cooperative Medical Scheme (NRCMS), a publicly subsidized health insurance programme in rural China, encourage individuals to engage in risky health behaviours	Cross- sectional study	New Rural Cooperative Medical Scheme (NRCMS) versus uninsured	NRCMS is a publicly subsidized health insurance programme in rural China	2000–2009	28 642	≥ 18		Good
**Sadaran-** **Gani *et al.*** ^38^	United States	To compare overall CVD risk and explore the importance of health insurance coverage on CVD risk relative to other health access barriers, from 2007 to 2012, in recent and long-term immigrants > 50 years of age	Cross- sectional study	Insured versus uninsured	Covered by any type of health insurance	2007–2012	1920	>50	54% (Recent immigrants) 53% (Long-term immigrants)	Good
**Stanciole** ^19^	United States	To estimate a structural model of an individual’s choice of insurance coverage and four lifestyle decisions: heavy smoking, heavy drinking, lack of exercise and obesity.	Cross- sectional study	Insured versus uninsured		1999–2003	5126	≥ 17		Medium
**Treviño** ***et al.*** ^53^	United States	To evaluate the centre for Medicare and Medicaid Services on intensive behaviour therapy for obesity program	Retrospect- ive cohort study	Medicare versus Medicaid versus county versus Private plan versus uninsured	Government- assistance health insurance (Medicare, Medicaid, county) and private plan	May 2012–February 2015	643	45–64	71.1	Medium
**Zhu *et al.*** ^33^	United States	To examine survey data from before and after California expanded its Medicaid program under the Affordable Care Act.	Cross- sectional study	Medicaid versus uninsured versus Private insurance versus Other coverage	Medicaid includes all those enrolled in Medicaid, even if they also had other insurance privately purchased it.	1997− 2013	514,043	18–65		Good
**Wilper** ***et al*.**^36^	United States	To explore whether insured Americans with three chronic conditions were less likely than the insured to be aware of their illness	Cross- sectional survey	Insured versus uninsured		1999–2006	41 510	18–64		Good

**Table 2:** Health insurance types and key findings

Author	Types of health insurance				Key findings
	Private	Community based	Public	Others/ Remarks	
Aqtash and Servellen^17^				Not specified	Health insurance is the only demographic variable that predicted health-promoting lifestyle behaviours (HPLB) (*t* = 1.99 [152], *P* = 0.049) among Arab immigrants. HPLB includes physical activity, stress management, health responsibility, nutrition, spiritual growth and interpersonal relations
Axelrod *et al.*^35^				Any health insurance	Individuals without insurance had a significantly higher HbA1c (6.1%, 43 mmol/mol) and BMI (30.0 ± 5.5 kg/m^2^) than those with insurance HbA1c (5.9%, 41 mmol/mol) and BMI (28.8 ± 5.4 kg/m^2^), *P* = 0.031 and *P* = 0.02, respectively.
Bittoni, *et al.*^39^	—				A greater proportion of the private insured reported a smoking history than the public/uninsured (58% versus 53%, *P* < 0.0001), while public/uninsured smokers have slightly more pack-years smoked. Mean BMI was slightly higher for the privately insured group (*P* < 0.0001). The privately insured group had a greater proportion of individuals classified with normal CRP levels < 3 mg/L (66% versus 57%; *P* < 0.0001), but also had significantly higher overall mean serum CRP levels (6.0 versus 4.5; *P* < 0.0001). Overall, elevated CRP, smoking, reduced diet quality and higher BMI were more prevalent in those with public insurance and were associated with increased risks of cancer/chronic disease mortality
Castillo-Laborde *et al.*^40^	—				Sixteen out of the 18 studied risk factors and disease prevalence were lower among private insurance beneficiaries, although the differences were insignificant after adjusting for age.
Dave and kaestner^20^			—	Medicare	Being a Medicare beneficiary was associated with a 24% decrease in the likelihood of engaging in physical activity. This increased to 40% when controlled for doctor visits. Medicare coverage is associated with a decrease in the number of cigarettes consumed daily without controlling for doctor visits. The association was small but statistically significant. (1.3%) Medicare is associated with a 31.8% (or 11.6% point) increase in the tendency for alcohol consumption among Medicare beneficiaries after controlling for doctor visits. Although this association was not statistically significant. In general, health insurance increased unhealthy lifestyles among the elderly.
Decker, *et al.*^32^			—	Medicaid	The uninsured adults were less likely to have diabetes, hypertension, or hypercholesterolemia (30.1% [95%CI, 26.8%–33.4%], compared with Medicaid recipients (38.6% [95%CI, 32.0%–45.3%]) (*P* = 0.02) and less likely to have multiple health conditions by 15.1% points [95%CI, 9.2%–20.9%, *P* <0.001]) Uninsured adults were less likely to be obese and sedentary than Medicaid recipients; however, binge drinking was more common among uninsured Overall, uninsured adults reported better health, fewer functional limitations and were less likely to have every specific health condition reported as compared with Medicaid beneficiaries
Dong *et al*.^50^			—	Urban Resident Basic Health Insurance (URBMI)	The probability of sedentariness increased significantly by 5.1% for individuals with URBMI than individuals with the Urban Employee Basic Medical Insurance. Being a URBMI enrollee increased the likelihood of smoking by 1.2%, but this is not statistically significant. The probability of using preventive care services, drinking alcohol/soft drinks, engaging in physical activity, and being overweight did not change significantly for URBMI insured Overall, URBMI did not change the utilization of preventive health care services, smoking/drinking habits, or other risky behaviours. However, the likelihood of being sedentary increased by 5% points.
Gotanda *et al.*^37^			—	Medicaid	Affordable Care Act’s (ACA)-Medicaid expansion was associated with lower systolic blood pressure but no evidence of change in diastolic blood pressure. ACA-Medicaid expansion was also associated with a lower HbAIc level. No evidence of change in the cholesterol levels after ACA-Medical aid expansion. In conclusion, there was improvement in two out of three cardiovascular risk factors accessed.
Hughes *et al.*^31^	—		—	HMOs and Medicare.	The percentages of participants engaged in lifestyle-related risks such as inadequate fruit/vegetable intake and physical activity were higher at 77.1% and 49.0% receptively among the insured. Heavy drinking (5.5%) and not receiving cervical cancer screening (8.5%) had a lower prevalence.
Jerant *et al.*^22^	—		—	Medicare Medicaid & Private	Gain or loss of health insurance was not associated with any significant change in health behaviours.
Kim *et al.*^51^			—	Medical aid & National Health Insurance	The brachial-ankle pulse wave velocity values were significantly higher among Medical aid beneficiaries than the National Health Insurance beneficiaries (1966 ± 495 versus 1582 ± 346 cm/s, *P* < 0.001).
Mahoney *et al.*^52^	—		—	Medicaid, Private & others	Possessing private health insurance was associated with a 0.82 (95%CI, 0.67–0.99) decrease in odds for the ratio of undiagnosed diabetes. Participants with no health insurance had the lowest prevalence of obesity compared with those with private health insurance, Medicaid or other types of health insurance (39.3% versus 43.1 and 53.7% and 48.3%, respectively)
Malta *et al.*^34^	—				There was an increase of 1.01% per year in free-time physical activities among the insured. Smoking prevalence decreased from 12.4% to 8.6% within a year among the population with private health insurance. There was equally higher alcohol consumption for both insured and uninsured groups. The consumption of fruits, legumes and vegetables increased at 0.72% per year. The frequency of overweight and obesity increase by 1.53% and 0.95% per year, respectively, among the population with private health insurance. No change in the prevalence of hypertension and diabetes among insured and uninsured but hypertension and diabetes were more frequent among people without private health insurance than the uninsured or those with other forms of insurance. In general, the prevalence of risk factors was lower and the frequency of protective factors higher among privately insured compared with the uninsured.
Malta *et al.*^29^				Not specified	There was a decrease of 12.4 to 7.7% (*P* < 0.00) in the number of smokers from 2008 to 2015 among individuals with health insurance. Overweight increased from 45.8 to 51.6% while obesity increased from 12.9 to 16.5% (*P* < 0.00). Recommended daily consumption of fruits and vegetables improved (24.9 to 30.9%; *P* < 0.00), but intake of excess fatty meat and consumption of beans did not change. Regular consumption of soft drinks (five or more times a week) dropped. Sufficient physical activity during leisure time increased from 35 to 43.9% (*P* < 0.00). There was no change in sedentary and alcohol consumption indicators and driving after drinking. The trend in hypertension did not change, but diabetes increased from 6% to 6.7% (*P* < 0.01)
Malta *et al.*^16^				Not specified	The participants with health insurance had higher prevalence of protective factors, such as fruit and vegetable intake (PR = 1.3 (95%CI, 1.2–1.3)), free time physical activity, (PR = 1.2 (95%CI, 1.2–1.3)), mammographies (RP =1.2 (95%CI, 1.1–1.3)) and pap smears (PR = 1.1 (95%CI,1.2–1.3)). Insured participants also had lower prevalence of risk factors such as smoking (RP = 0.7(95%CI, 0.6–0.8)), poor health (RP = 0.8 (CI95%, 0.6–0.9)), obesity (RP = 0.8 (95%CI, 0.7–0.9)), consumption of meat with fat (RP = 0.9 (95CI%, 0.8–0.9)) and whole milk (RP = 0.9 95%CI 0,8–0.9)). The prevalence of self-reported hypertension and diabetes was less among the population with health insurance than those without health insurance.
Malta and Berna^28^				Not specified	Compared with the uninsured, health insurance beneficiaries were more likely to have protective factors, such as healthy eating, physical activity, coverage (mammography and Pap test), and lower risk factors such as smoking, physical inactivity, poor health assessment, and hypertension. Alcohol abuse, overweight, obesity, excessively fatty meats consumption and diabetes were not associated with health insurance status. Participants with health insurance generally have better indicators
Miraldo *et al*.^21^			—		Increasing public subsidized health insurance did not affect behaviour risk factor or diabetes disease management except for alcohol intake in the lower-income white participant’s subgroup.
Mpofu *et al.*^18^	—	—		Uninsured- govern-ment free, public/ national health system only	Compared to women with health insurance, women without health insurance were more likely to report physical inactivity (aRR = 1.1 (1.1–1.2)), smoking (aRR = 1.4 (1.1–1.8)), and self-reported hypertension aRR = 1.4 (1.1–1.7))
Qin and Lu^30^			√		Rural Cooperative Medical Scheme (NRCMS) coverage in China decreased the probability of being sedentary at 95.1% versus 96% in the uninsured group. NRCMS increased the probability of smoking by 1.9%. (significant at the 10% level). Participating in NRCMS increased the probability of smoking by 1.9%. Being NRCMS participants encourage a daily high-calorie intake of 2180 kcal versus 2179.1 kcal in non-participants and decrease the percentage of healthy energy food consumption. NRCMS participants have a significantly higher tendency to be overweight than non-participants (27.4% versus 22%).
Sadarangani *et al.*^38^				Any health insurance	Being insured contributed more to cardiovascular risk than other factors relating to health care access; it also increased the likelihood of being at risk of cardiovascular disease by 1.7 folds.
Stanciole^19^				Any health insurance	The insured tend to smoke more with a prevalence of 5% compared to 3% in the uninsured sample, drink more alcohol (8% versus 4%) and tend to be more sedentary (16% versus 11%). The two groups fare very similarly in the prevalence of obesity (25% versus 26%). The insured had a higher prevalence of stroke, high blood pressure, diabetes, cancer, heart attack and coronary heart disease, arthritis and loss of mental ability.
Treviño *et al.*^53^	—		—	Medicare, Medicaid, county & Private	BMI and weight decreased irrespective of insurance status and demographic profile such as age sex, sex, race or health status. Participants lost 0.102 kg per session attended on the average
Zhu *et al.*^33^	—		—	Medicaid & Private plan	There was a decrease in the unadjusted smoking prevalence rate irrespective of insurance status, from 33.8% to 27.8% for Medicaid recipients, 38.6% to 29.3% for the uninsured group, 21.3% to 13.7% for the privately insured and 22.6% to 15.0% for those with other types of insurance coverage. Smokers in Medicaid were more likely to have a chronic disease (55.0%) as compared with those with Private Insurance (37.3%, *P* < 0.01) or those without Insurance (32.4%, *P* < 0.01).
Wilper *et al*.^36^				Any health insurance	In general, undiagnosed and uncontrolled chronic illness was more frequent among the uninsured when compared to the insured. Although undiagnosed hypertension was not associated with insurance status, the uninsured were more likely to have uncontrolled hypertension compared with insured individuals (42.0% versus 35%, *P* = 0.03)

### Data synthesis

The reports were classified into three: (i) HI and healthy lifestyle: (a) physical activities, (b) smoking, (c) alcohol consumption and (d) healthy diets, (ii) HI and health outcomes related to NCDs and (iii) private HI versus other forms of HI status. We considered three possible effects: positive, negative and no effects. The heterogeneity of the studies included in this review precluded a formal meta-analysis.

## Results

**Fig. 1:**
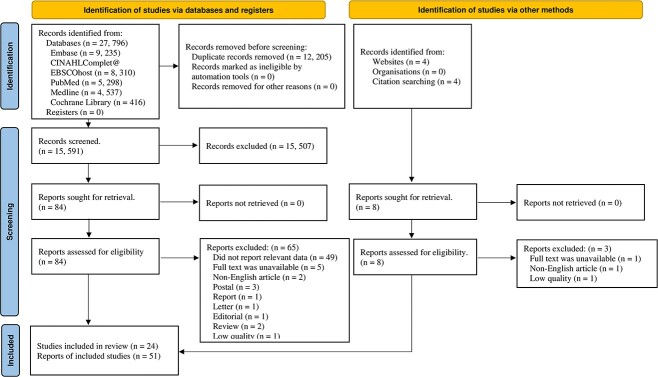
PRISMA diagram (Flow of article search, screening, article assessment for exclusion and inclusion criteria with reasons for exclusion detailed).

A total of 27 796 articles were identified from the initial search. A total of 12 205 articles were excluded after screening for duplicates. A total of 15 591 articles were screened for relevance through their titles and abstracts, leaving 84 articles for further in-depth review. A total of 65 articles were excluded for various reasons, leaving 19 eligible articles, as shown in Figure 1. Eight additional articles were identified through other sources. Three articles were excluded for various reasons. The total eligible article was 24 (6 longitudinal and 18 cross-sectional studies). The summary characteristics of the included articles can be found in Table 1.

### Health insurance and health lifestyle

#### Effect of health insurance on physical activities

Eleven out of the 24 studies reported the effect of HI coverage on engaging in physical activities, comparing the insured with the uninsured. More reports (6/11 studies) claimed HI encouraged physical activity engagement.^17^^,^^18^^,^^21^^,^^28–30^ On the other hand, some studies^19^^,^^20^^,^^31^^,^^32^ indicated that HI contributed to physical inactivity or the likelihood of adopting a sedentary lifestyle. One study by Miraldo *et al.*^21^ reported that HI has no effect on physical activity or the propensity to be sedentary.

#### Effect of health insurance on smoking

Eleven studies reported the effect of HI on smoking in comparative studies between the insured and uninsured. Five of these reports claimed HI reduces the propensity to smoke.^16^^,^^18^^,^^20^^,^^28^^,^^29^ Based on the studies by Malta and Bernal ^28^ and Malta *et al*.^16^ in Brazil, HI beneficiaries had a lower prevalence of smoking participants. Malta *et al*.^29^ also reported a positive decrease in smoking prevalence among insured participants in Brazil. Dave and Kaestner^20^ found that being a Medicare beneficiary is associated with a decrease in the number of cigarettes consumed daily without controlling for doctors’ visits. The association was relatively small (1.3%) but statistically significant. The observation by Qin and Lu^30^ was relatively different; they found that participating in the New Rural Cooperative Medical System (NRCMS) increased the probability of smoking by 1.9%. This is in line with the report by Stanciole.^19^ Four other reports claimed HI has no effect on the choice to smoke.^21^^,^^22^^,^^33^ In conclusion, more reports concluded that HI reduced the propensity to smoke as against no effect or increased effects.

#### Effect of health insurance on alcohol consumption

Ten studies examined the link between HI and alcohol consumption among insured and uninsured participants. Five of the 10 studies claimed HI did not encourage alcohol consumption,^16^^,^^19^^,^^30–32^ while one study claimed HI is associated with increased alcohol consumption.^20^ Four of the studies claimed HI does not affect alcohol consumption.^21^^,^^28^^,^^29^^,^^32^ Stanciole,^19^ in his report, stated that HI decreases the tendency for heavy drinking, while Decker *et al*.^32^ found binge drinking more common among uninsured participants. Malta *et al***.**^16^ claimed alcohol use was at 17% (95%CI, 16.1–18.0) among people with HI but slightly higher among the uninsured group. In general, more reports concluded that HI has a positive effect on alcohol consumption as against negative or no effect.

#### Effect of health insurance on healthy diets

Seven studies examined the relationship between HI and diet among people with and without HI. Four of these studies suggested a positive link.^16^^,^^17^^,^^29^^,^^34^ The result published by Malta *et al.*^29^ showed a positive increasing trend in fruit and vegetable intake. Malta *et al.*^16^ found more frequent healthy diet indicators and less frequent unhealthy diet indicators in participants with HI than those without HI. The healthy diet indicators reported include sufficient intake of fruits and vegetables, which was at 30.9% (95%CI, 29.8–32.1) versus 19.8% (95%CI, 18.7–20.9) for the insured and uninsured, respectively. Two studies claimed HI is associated with poor diet.^30^^,^^31^ Qin and Lu^30^ found that being NRCMS participants encouraged a daily high-calorie intake of 2180 kcal versus 2179.1 kcal in non-participants and decreased the percentage of healthy energy food consumption. One study claimed HI has no effect on diet.^21^ Overall, more reports concluded that HI encouraged a healthy diet.

### Health insurance and health outcomes

Nine studies reported the impact of HI on health outcomes. Six of the nine reports suggest that HI coverage is associated with the lowered prevalence of the assessed NCDs.^16^^,^^18^^,^^28^^,^^35–37^ Two reports indicated that HI has a negative effect on measured health comes^32^^,^^38^ while one report states that HI has no effect on hypertension.^29^ The report by Wilper *et al*.^36^ stated that undiagnosed and uncontrolled chronic illness was more frequent among the uninsured when compared to the insured. Although undiagnosed hypertension was not associated with insurance status. Sadarangani *et al*.^38^ found that being insured contributed more to cardiovascular risk than other factors relating to healthcare access; it also increased the likelihood of being at risk of cardiovascular disease by 1.7 fold. In all, more reports associated HI with lowered prevalence of NCDs.

### Private health insurance versus other forms of health insurance status

Three studies compared the effect of private HI schemes against other forms of HI on health behaviours or outcomes. All three studies reported better health outcomes and (or) behaviours with private HI compared to other insurance schemes.^33^^,^^39^^,^^40^ The study by Bittoni *et al.*^39^ found that smoking, poor diet and raised C-reactive protein were more prevalent in people with public HI compared to the privately insured. Castillo-Laborde *et al*.^40^ found that risk factors and disease prevalence were lower among people with private HI.

## Discussion

### Main findings

Twenty-four evaluations of the effect of HI on healthy lifestyle choices and (or) the presence of NCDs were identified. The results from these studies are mixed. However, most reports from these evaluations indicated HI encouraged healthy lifestyle choices. The most common health status measured are hypertension and diabetes. More reports concluded that HI was associated with a lowered prevalence of NCDs. Three evaluations on private HI were identified. All the evaluations found that private HI encouraged a healthy lifestyle. Furthermore, most of the studies on the effect of HI are cross-sectional-based.

As stated earlier, the effect of HI on health-related outcomes are mixed, although most studies indicate that HI is beneficial. The variation in results could be related to the process of recruiting participants for the study. There is a high possibility of sampling bias since some HI coverage is guided by eligibility and availability, such as the Medicare scheme under the Affordable Care Act in the United States of America (USA).^32^ Similar to this is the NRCMS, a HI scheme subsidized by the government for rural residents in China.^30^ Hence, the samples pulled from the enrollee of the insurance scheme will not be a perfect representation of the population. The effect of HI from this sample is expected to differ from another study group where insurance enrolment is based on other eligibility criteria. Co-founding variables is another factor that could alter the measured impact or effect of HI on health-related outcomes. The presence of a co-founder could lead to under or over-estimation of the effect of HI. Dave and Kaestner^20^ showed that the effect of being a Medicare beneficiary on physical activity increased when controlled for doctor visits. The effect of confounding variables should be vividly considered (if measurable) and reported when assessing the effect of HI.

HI is reported to have varying effects based on the country of study. In Brazil, people with HI coverage mostly make healthy lifestyle choices which are relatively different from the observation in the USA. The difference in observations in the USA and Brazil is not surprising, as both countries’ healthcare systems differ. The USA has a less cost-effective multiplayer system when compared with its neighbouring Canada’s single-payer system.^41^

The USA HI industry is dominated by private HI schemes, which cover about 50% of Americans.^42^ The private HI schemes in the USA offer different premiums and packages^43^ and most people get it from their employers and are sponsored by co-payment by both the employer and employee.^42^ The government, however, provides publicly funded insurance schemes for the elderly and people in the low-income group, known as Medicare and Medicaid, respectively, based on eligibility.^42^ Hence, the HI system in the USA is very competitive and beneficiaries are propelled to maximize their insurance benefit. This could potentiate extra-moral hazards, where insurance beneficiaries tend to lose personal inducement to engage in a healthy lifestyle in lieu of HI benefits.

Unlike the USA, the Brazilian healthcare system is dominated by the public HI industry, similar to the healthcare system in Canada and the UK.^43^ Brazil has the largest populace with broad healthcare coverage globally, without incurring ‘out-of-pocket’ expenditure on healthcare users.^44^ This is achieved through the Sistema Único de Saúde, the unified health system launched in 1991.^45^ Other HI plans in Brazil include private HI, prepaid group practice and company health plans.^18^ Although most of the Brazilian population is covered under the Unified health system,^46^ some people (those that can afford it) still opt for personal insurance. As of 2013, about 27.9% of Brazilians had private insurance plans.^47^ This is because the health system is faced with a shortage of resources^48^ and enrollee would prefer personal HI to ensure timely access to healthcare.

The type of insurance determines the effect of HI. In most cases, private HI increases the likelihood of making a healthy lifestyle choice as opposed to other schemes. Although the articles reviewed in this study did not identify if the private HI schemes had integrated packages to induce healthy lifestyle choices. There are instances where HI providers promote healthy lifestyles among their clients by encouraging physical activity. This is mostly achieved by providing fitness centre offers. Furthermore, some insurers provide mobile devices to facilitate easy monitoring of diet, physical activity or sleep patterns. Clients who hit the mark of healthy living are rewarded monetarily with premium discounts or more. This is to improve clients’ health and lower medical costs.^49^ This also serves as a check on ex-ante moral hazard.

Determining the effect of HI at a global level might be challenging. Accessing HI nationally is more feasible since most countries have unique HI structures.

### What is already known on this topic

HI has varying effects on healthy lifestyle choices. This either encouraged healthy lifestyle choices, increased the propensity to engage in unhealthy lifestyles, or had no effect on an individual’s tendency to make healthy lifestyle choices. More also, there are limited studies of this context in developing countries.

### What this study adds

Most reports from this evaluation indicated that HI encouraged healthy lifestyle choices and was associated with a lowered prevalence of NCDs. The most common health outcomes measured were hypertension and diabetes. All the evaluations found that private HI was associated with better health outcomes and (or) healthy lifestyle choices. Furthermore, most of the studies on the effect of HI were cross-sectional-based.

### Limitations of this study

One of the limitations of this review is the possibility that some studies were missed out, as papers published in languages other than English were not sorted. Three non-English studies were identified and excluded in this comprehensive database search. Secondly, unpublished articles were excluded as they might not have the same methodological quality as published data. On the third note, there are limited studies at the national level for each outcome, making it impossible to determine the effect of HI at the country level.

## Conclusion

In summary, HI is viewed as a tool used to increase healthcare access. Nonetheless, the link between HI and lifestyle choices has not been established. This study identified more reports in favour of the positive effect of HI on healthy lifestyle choices and the prevalence of NCDs. In general, this evidence suggests that HI is more beneficial, while private HI seems more effective in facilitating health protection than other healthcare plans. The review has contributed more data on the role of HI in healthy lifestyle choices and its contribution to the presence of NCDs. This has also provided information for further research in this field.

## Supplementary Material

Appendix_A_fdad247

Appendix_B_fdad247

Appendix_C_fdad247

Appendix_D_fdad247

Supplementary_information_fdad247

PRISMA_2020_checklist_2f_fdad247

## Data Availability

The studies included in the review will be available upon request.

## Notes

**Adeola Folayan,** PhD student

**Mark Wing Loong Cheong,** Senior Lecturer

**Quek Kia Fatt,** Associate Professor

**Tin Tin Su,** Professor
