# Supplementary material for: Health insurance status, lifestyle choices and the presence of non-communicable diseases: a systematic review
Source: J Public Health (Oxf). 2023 Dec 11;46(1):e91–e105. doi: 10.1093/pubmed/fdad247 (PMC10901270; doi:10.1093/pubmed/fdad247)
Supplement: Appendix_A_fdad247 [file appendix_a_fdad247.docx]

# Appendix A: Search strategy

(“health Insurance” OR “healthcare insurance” OR “ insurance status” OR “medically insured” OR “national health program” OR “insurance coverage” OR "medicare" OR “medicaid” OR “health services accessibility” OR “Community based insurance” OR “medical security” OR “medical insurance” OR “public insurance” OR “healthcare financing” OR “health financing”) AND (“Healthy Lifestyle” OR “Healthy Life style” OR “Health Behavior” OR “Health Promotion” OR “Obesity” OR “Ex-ante moral hazard” OR “Propitious selection” OR “Prevention activity*” OR “Physical activity*” OR “Physical inactivity*” OR “Exercise*” OR “Sedentary Behavior” OR “Sport*” OR “Nutrition “ OR “Diet” OR “Eating” OR “Feeding” OR “Vegetable consumption” OR “Smoke*” OR “Alcohol consumption” OR “Alcohol use” OR “Alcohol drinking” OR “Alcohol intake” OR “Drinking habit”) AND (“Non-communicable Disease” OR “Noncommunicable Disease” OR “NCD” OR “Cardiovascular disease” OR “Heart Disease” OR “Hypertension” OR  “High blood pressure” OR “Heart attacks” OR “Myocardial infarction” OR “ Rheumatic heart disease” OR  “Bouillaud* disease” OR “Cardiomyopathy” OR “Atrial fibrillation” OR “ Endocarditis” OR “Aortic aneurysms” OR “Peripheral artery disease” OR “Peripheral arterial diseases” OR “Peripheral vascular disease” OR “Peripheral angiopathy*” OR “Coronary heart disease” OR “Coronary disease ” OR “Heart failure” OR “Myocardial failure” OR “ Cardiac failure” OR “Cerebrovascular disease” OR “ Vascular disorder“ OR “Intracranial vascular disease” OR “Cerebrovascular disorder” OR “Cardiomyopathies” OR “Myocardial disease” OR “Deep vein thrombosis” OR “Pulmonary embolism” OR “Ischem*” OR “atherosclero*” OR “arteriosclero*” OR “Diabetes Mellitus” OR “Glucose intolerance” OR “Chronic respiratory diseases” OR “CRD” OR “Pulmonary hypertension” OR “Asthma” OR “Chronic obstructive pulmonary disease” OR “Chronic airflow obstruction” OR “Chronic obstructive airway disease” OR “COPD” OR “Lung diseases” OR “Cancer” OR “Casino*” OR “Malignancies” OR “Malignancy” OR “Neoplasia” OR “Neoplasm” OR “Tumor”
