# Supplementary material for: Health insurance status, lifestyle choices and the presence of non-communicable diseases: a systematic review
Source: J Public Health (Oxf). 2023 Dec 11;46(1):e91–e105. doi: 10.1093/pubmed/fdad247 (PMC10901270; doi:10.1093/pubmed/fdad247)
Supplement: Appendix_B_fdad247 [file appendix_b_fdad247.docx]

# Appendix B: Quality Assessment Tools

## -Critical Appraisal Checklist for survey-based studies [Center for Evidence Based Management]

# Did the study address a clearly focused issues/question?

# 2. Is the research method (study design) appropriate for answering the research question?

# 3. Is the method of selection of the subjects (employees, teams, divisions, organisations) clearly described?

# 4. Could the way the sample was obtained introduce (selection) bias? 5. Was the sample of subjects representative with regard to the population to which the findings will be referred?

# 6. Was the sample size based on pre-study considerations of statistical power?

# 7. Was a satisfactory response rate achieved?

# 8. Are the measurements (questionnaires) likely to be valid and reliable?

# 9. Was the statistical significance assessed?

# 10. Are confidence intervals given for the main results?

# 11. Could there be confounding factors that haven’t been accounted for?

# 12. Can the results be applied to your organisation?

## -The NIH Quality Assessment Tool for Observational Cohort and Cross-Sectional

# 1. Was the research question or objective in this paper clearly stated?

# 2. Was the study population clearly specified and defined?

# 3. Was the participation rate of eligible persons at least 50%?

# 4. Were all the subjects selected or recruited from the same or similar populations (including the same time period)? Were inclusion and exclusion criteria for being in the study prespecified and applied uniformly to all participants?

# 5. Was a sample size justification, power description, or variance and effect estimates provided?

# 6. For the analyses in this paper, were the exposure(s) of interest measured prior to the outcome(s) being measured?

# 7. Was the timeframe sufficient so that one could reasonably expect to see an association between exposure and outcome if it existed?

# 8. For exposures that can vary in amount or level, did the study examine different levels of the exposure as related to the outcome (e.g., categories of exposure, or exposure measured as continuous variable)?

# 9. Were the exposure measures (independent variables) clearly defined, valid, reliable, and implemented consistently across all study participants?

# 10. Was the exposure(s) assessed more than once over time?

# 11. Were the outcome measures (dependent variables) clearly defined, valid, reliable, and implemented consistently across all study participants?

# 12. Were the outcome assessors blinded to the exposure status of participants?

# 13. Was loss to follow-up after baseline 20% or less?

# 14. Were key potential confounding variables measured and adjusted statistically for their impact on the relationship between exposure(s) and outcome(s)?
