# Supplementary material for: Health insurance status, lifestyle choices and the presence of non-communicable diseases: a systematic review
Source: J Public Health (Oxf). 2023 Dec 11;46(1):e91–e105. doi: 10.1093/pubmed/fdad247 (PMC10901270; doi:10.1093/pubmed/fdad247)
Supplement: Appendix_C_fdad247 [file appendix_c_fdad247.docx]

**Appendix C: Quality assessment procedure**

A critical appraisal checklist for survey-based studies was used to assess the quality of the selected studies. The checklist contains 12 questions. Each question was scored as either reported or not reported. The question was scored one if reported and zero if not reported. The final selection was based on the result of the quality assessment. Studies with 7–12 points were graded as good quality, while studies with 4–6 points were graded as medium quality. Studies with less than 4 points were considered low quality. Only good and medium quality studies were included in the study.  The NIH Quality Assessment Tool for Observational Cohort and Cross-Sectional Studies was used to assess the longitudinal studies. The NIH Quality assessment has 14 questions. The question is scored one if reported appropriately and zero if the answer cannot be determined or not reported.  Studies with 11-14 points were graded as good quality, while studies with 5-10 points were graded as fair quality. Studies with 4 points or less were considered poor quality. Only good and fair quality (medium) studies were included in the study. The two assessment tools are attached in Appendix B. The assessment was done by one reviewer (AF) and validated by another reviewer (MC). The two reviewers discussed discrepancies in the studies. Discrepancies were resolved through consultation with the third reviewer (TTS).
