# Supplementary material for: Health insurance status, lifestyle choices and the presence of non-communicable diseases: a systematic review
Source: J Public Health (Oxf). 2023 Dec 11;46(1):e91–e105. doi: 10.1093/pubmed/fdad247 (PMC10901270; doi:10.1093/pubmed/fdad247)
Supplement: Supplementary_information_fdad247 [file supplementary_information_fdad247.docx]

**Supplementary information**

**Funding:** None

**Competing interests:** None declared.

**Ethical Approval and Consent to participate:** Not applicable

**Consent for publication:** Not applicable

**Availability of supporting data:** The studies included in the review will be available upon request.

**Authors' contributions:** TTS conceptualised the review. All authors contributed to the study design. Title and abstract screening were carried out by AF and MC. AF wrote the first draft, which was edited and approved by all authors.

**Acknowledgements:** Not applicable

**Authors' information**

AF is a PhD student, South East Asia Community Observatory (SEACO), Jeffrey Cheah School of Medicine & Health Sciences, Monash University Malaysia.

MC is a Senior Lecturer, School of Pharmacy, Monash University Malaysia

# QKF is an Associate Professor in Community Health, Jeffrey Cheah School of Medicine & Health Sciences, Monash University Malaysia

# TTS is a Professor of Public Health, Deputy Director, South East Asia Community Observatory (SEACO), Jeffrey Cheah School of Medicine & Health Sciences, Monash University Malaysia
